# The nuclear receptor NOR-1 regulates the small muscle protein, X-linked (SMPX) and myotube differentiation

**DOI:** 10.1038/srep25944

**Published:** 2016-05-16

**Authors:** Beatriz Ferrán, Ingrid Martí-Pàmies, Judith Alonso, Ricardo Rodríguez-Calvo, Silvia Aguiló, Francisco Vidal, Cristina Rodríguez, José Martínez-González

**Affiliations:** 1 Centro de Investigación Cardiovascular, CSIC-ICCC, IIB-Sant Pau, c/Sant Antoni Maria Claret 167, 08025 Barcelona, Spain; 2 Unitat de Diagnòstic i Teràpia Molecular, Banc de Sang i Teixits, Passeig Vall d’Hebron 119-129, Barcelona 08035, Spain

## Abstract

Recent works have highlighted the role of NOR-1 in both smooth and skeletal muscle, and have proposed this nuclear receptor as a nexus that coordinates muscle performance and metabolic capacity. However, no muscle specific genes regulated by NOR-1 have been identified so far. To identify NOR-1 target genes, we over-expressed NOR-1 in human vascular smooth muscle cells (VSMC). These cells subjected to sustained over-expression of supraphysiological levels of NOR-1 experienced marked phenotypic changes and up-regulated the skeletal muscle protein X-linked (SMPX), a protein typically expressed in striated muscle and associated to cell shape. By transcriptional studies and DNA-protein binding assays, we identified a non-consensus NBRE site in human SMPX promoter, critical for NOR-1 responsiveness. The expression of SMPX was higher in human skeletal muscle myoblasts (HSMM) than in human VSMC, and further increased in HSMM differentiated to myotubes. NOR-1 silencing prevented SMPX expression in HSMM, as well as their differentiation to myotubes, but the up-regulation of SMPX was dispensable for HSMM differentiation. Our results indicate that NOR-1 regulate SMPX in human muscle cells and acts as a muscle regulatory factor, but further studies are required to unravel its role in muscle differentiation and hypertrophy.

Neuron-derived orphan receptor-1 (NOR-1) is a transcription factor belonging to the Nuclear Receptor subfamily 4 group A (NR4A). NOR-1 was firstly identified in forebrain neural cells undergoing apoptosis^1^; however, results from different cell types support a role for this nuclear receptor in an array of tissues, modulating diverse and sometimes antithetical processes such as cell survival and apoptosis, and cell proliferation and differentiation^2^,^3^,^4^,^5^. NOR-1 participates in vascular biology, inflammation, immunity, and in lipid and glucose metabolism^6^,^7^,^8^,^9^,^10^. In fact, NOR-1 misregulation has been associated with a variety of high-incidence human pathologies including cardiovascular disease, diabetes, obesity and cancer^3^,^6^,^7^,^8^,^11^.

Structural studies have shown that NOR-1, as well as the other two members of the NR4A family (Nur77 and Nurr1), lacks an accessible ligand-binding pocket^12^,^13^. These receptors seem to be constitutively active and their transcriptional activities are mainly dependent on their expression levels. NOR-1 is expressed at low levels in vascular tissues and resting vascular cells, but it is quickly induced by growth factors and cytokines, acting as an immediate-early response gene involved in vascular hyperplasia^4^,^14^,^15^,^16^,^17^,^18^,^19^. NOR-1 is also expressed in the heart and skeletal muscle (at high levels compared with other organs)^4^,^20^,^21^,^22^, and in these tissues NOR-1 up-regulation has been associated with hypertrophy^23^,^24^. Therefore, both the basal expression of NOR-1 and the pathophysiological consequences of NOR-1 over-activation seem to be tissue-dependent. Gain- and loss-of-function studies have allowed to identify a few NOR-1 target genes involved in vascular cell proliferation (e.g. cyclin D1 and S phase kinase-associated protein 2 [SKP2])^17^,^25^, and have associated this receptor with the expression of genes involved in specific aspects of lipid, carbohydrate and energy homeostasis in striated muscle^23^,^26^,^27^. However, today a limited number of NOR-1 target genes have been reported. In the present study, we over-expressed this NR4A receptor in human vascular smooth muscle cells (VSMC) by lentiviral transduction to identify new NOR-1 target genes. VSMC subjected to sustained over-expression of supraphysiological levels of NOR-1 experienced marked phenotypic changes and up-regulated a gene typically expressed in striated muscle (skeletal muscle protein X-linked, SMPX). We characterized the regulation of SMPX by NOR-1 and uncovered the critical role of this nuclear receptor in the differentiation of human skeletal myoblasts to myotubes, a process which also entails the up-regulation of SMPX.

## Results

### NOR-1 over-expression in VSMC modifies cell shape and increases SMPX expression

**Figure 1:**
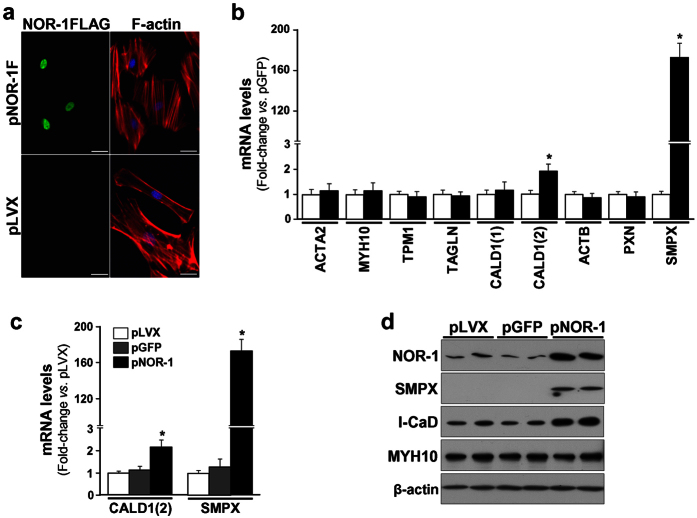
Lentiviral NOR-1 over-expression induces phenotypic changes in human VSMC and up-regulates SMPX. VSMC were transduced with lentiviral vectors to over-express NOR-1 (pLVX/NOR-1; pNOR-1), a FLAG-tagged derivative form of NOR-1 (pLVX/NOR-1-FLAG; pNOR-1F) or with control lentiviral vectors (pLVX or the EGFP expression vector [pLVX/EGFP; pGFP]). (**a**) Immunofluorescence microscopy analysis showing FLAG-tagged NOR-1 (green), filamentous actin (F-actin stained with phalloidin, red) and nuclei (Hoechst stain, blue). (**b**) Expression levels (analyzed by real-time PCR) of a panel of genes related to cell shape/morphology encoding for cytoskeleton and cytoskeletal-associated proteins in control cells (white bars) and cells transduced with pNOR-1F (black bars): ACTA2 (smooth muscle α-2 actin), MYH10 (myosin heavy chain 10), TPM1 (tropomyosin 1), TAGLN (transgelin), CALD1(1) (caldesmon transcript specific for smooth muscle cells), CALD1(2) (caldesmon transcript ubiquitously expressed, encoding for a light form of the protein [l-CaD]), ACTB (β-actin), PXN (paxillin) and SMPX (small muscle protein, X-linked) (n = 6). *P < 0.0001 *vs*. cells transduced with pGFP. (**c**) CALD1(2) and SMPX mRNA levels in VSMC transduced with pNOR-1 or with control lentiviral vectors. Data were normalized as in (b) (n = 6). *P < 0.0001 *vs*. cells transduced with pLVX. (**d**) Representative Western blot showing protein levels of NOR-1, SMPX, l-CaD, MYH10 and β-actin in VSMC over-expressing NOR-1 and control cells (n = 4).

Human VSMC were transduced to over-express NOR-1 (Supplementary Fig. S1). VSMC over-expressing NOR-1 exhibited striking differences in cell morphology compared to control cells (Fig. 1a). This phenotypic switch in cells expressing high and sustained levels of NOR-1 was associated to a low proliferation rate (Supplementary Fig. S2a) and signs of cell hypertrophy such as increased total cellular protein and enhanced cellular surface area (Supplementary Fig. S2b,c). As a first approach to identify genes regulated by NOR-1, we analyzed the mRNA levels of several genes related to cell shape/morphology encoding for cytoskeleton and cytoskeletal-associated proteins. The over-expression of NOR-1 did not alter the expression of most of these genes, but slightly induced the expression levels of the transcript corresponding to the ubiquitously expressed form of caldesmon (l-CaD; ≈ 1.9-fold). The most striking results, however, was the strong up-regulation of SMPX (a stretch-responsive skeletal muscle protein) (Fig. 1b). SMPX was up-regulated up to 170-fold as compared with control cells (transduced with pLVX or pLVX/EGFP) (Fig. 1c). The regulation of both SMPX and l-CaD was confirmed by Western blot (Fig. 1d). SMPX expression was detected at low levels in human vascular tissues (aorta and coronary arteries) compared to its expression in skeletal muscle (Supplementary Fig. S3).

### NOR-1 induces SMPX promoter activity

**Figure 2:**
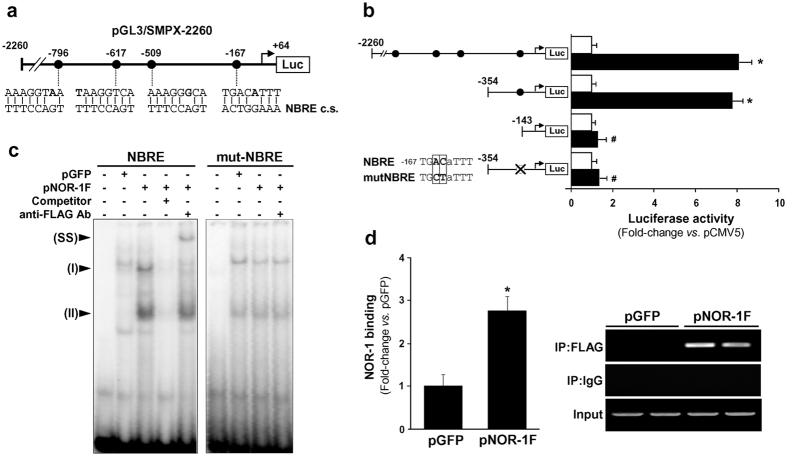
Human SMPX promoter activity is regulated by NOR-1. (**a**) Scheme showing the four putative NBRE elements present in the SMPX promoter region cloned into the pGL3 reporter vector (pGL3/SMPX-2260). The consensus NBRE sequence (NBRE c.s.) is indicated and non-conserved bases are shown in bold. (**b**) Luciferase activity from cells co-transfected with a NOR-1 expression vector (pCMV5/NOR-1; black bars) or the corresponding empty plasmid (pCMV5; white bars) together with different pGL3/SMPX constructs. The activity of constructs mutated in the NBRE site (deleted white circle) is also shown. The core of the NBRE(−167/−160) is indicated and changes introduced by mutagenesis on constructs are boxed (n = 6). *P < 0.0001 *vs*. cells co-transfected with pCMV5; ^#^P < 0.0001 *vs*. cells co-transfected with pCMV5/NOR-1 and SMPX constructs containing the native NBRE(−167/−160) site. (**c**) Representative autoradiograms of EMSA performed with a SMPX probe containing the NBRE(−167/−160) site (NBRE) and nuclear protein extracts from VSMC transduced with lentiviral vectors to express NOR-1-FLAG (pNOR-1F) or EGFP (pGFP). The position of the complexes up-regulated by NOR-1 (I and II) is indicated. Competition assays with a molar excess of unlabeled probe (100-fold; Competitor) and supershift assays with a specific antibody against the FLAG sequence (anti-FLAG Ab) were performed. EMSA carried out with a mutated NBRE probe (mut-NBRE) is also shown. SS: supershifted complex. (**d**) The relative *in vivo* association of NOR-1 with SMPX promoter was analyzed by ChIP assays in VSMC transduced with pGFP or pNOR-1F. Immunoprecipitations were performed with an antibody against the FLAG sequence (IP:FLAG) or a non-specific IgG (IP:IgG). The bars graph shows the enrichment of NOR-1 binding quantified by real-time PCR using SMPX promoter specific primers. Data were normalized to the total input DNA (n = 4). *P = 0.0002 *vs.* pGFP. The panel on right shows the agarose gel electrophoresis of PCR products. Equal input DNA and control IgG immunoprecipitations are shown.

While no NOR-1 response elements were detected in the promoter of the caldesmon encoding gene (CALD1)*, in silico* analysis revealed the presence of four putative NBRE binding sites in the SMPX promoter (positions −796, −617, −509 and −167 from the transcription start site), although none of them was fully conserved (Fig. 2a). To analyze the functionality of these elements, a promoter fragment (−2260 to +64) was cloned into the luciferase reporter plasmid pGL3. In co-transfection assays, the pCMV5/NOR-1 expression vector significantly induced SMPX transcriptional activity (Fig. 2b). The contribution of each putative NBRE to the NOR-1-dependent SMPX regulation was established by promoter deletion and site-directed mutagenesis. As shown in Fig. 2b, the deletion of a fragment containing the three distal putative NBRE sites did not affect the up-regulation of SMPX promoter elicited by NOR-1. The deletion of the proximal NBRE(−167/−160) site or its mutagenesis abrogated the NOR-1-mediated induction of SMPX transcriptional activity, suggesting that this site confers NOR-1-responsiveness. In contrast to human SMPX, mouse SMPX seems to be unresponsive to NOR-1, as far as no NOR-1 response elements could be detected in mouse SMPX promoter and neither regulation of SMPX by NOR-1 in mouse aorta nor in mouse VSMC was observed (Supplementary Fig. S4).

### NOR-1 binds to SMPX promoter through a non-consensus NBRE site

To further characterize the functionality of the putative NBRE(−167/−160) site, we performed electrophoretic mobility shift assays (EMSA) using nuclear extracts from VSMC over-expressing a FLAG-tagged NOR-1. As shown in Fig. 2c, a differential pattern of retarded bands was observed in EMSA using this NBRE site as a probe and nuclear extracts from NOR-1-transduced cells, respect those from control cells (transduced with pLVX/EGFP). NOR-1 over-expression increased the intensity of two of these complexes, that were competed by an excess of unlabeled probe, and one of them (complex I) was supershifted by an anti-FLAG antibody. Conversely, mutation of the NBRE probe abrogated both the enhanced binding found in NOR-1-transduced cells and the supershift. Taken together, these data demonstrate that NOR-1 specifically bind to this NBRE site. To confirm that NOR-1 actually binds to the SMPX promoter *in vivo*, chromatin immunoprecipitation (ChIP) assays were performed in VSMC over-expressing FLAG-tagged NOR-1. Proteins directly bound to chromatin were immunoprecipitated using an antibody against the FLAG sequence or an unspecific IgG. Conventional and real-time PCR were used to amplify a genomic region surrounding the NBRE(−167/−160) site in SMPX promoter. Consistent with data from EMSA, ChIP assays demonstrated that NOR-1 specifically binds to the SMPX promoter region encompassing the NBRE site (Fig. 2d). A control IgG did not precipitate detectable DNA and pre-immunoprecipitation samples evidenced equivalent DNA input.

### NOR-1 knockdown decreased SMPX mRNA and protein levels in human skeletal muscle myoblasts (HSMM)

**Figure 3:**
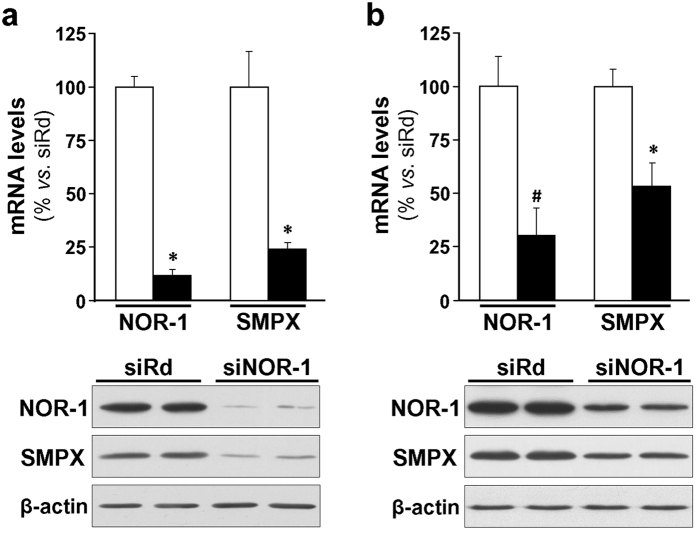
NOR-1 knockdown down-regulates SMPX expression. (**a**) HSMM were transfected with a siRNA against NOR-1 (siNOR-1; black bars) or a random siRNA (siRd; white bars). The expression of NOR-1 and that of SMPX in these samples was analyzed by real-time PCR (upper panel) (n = 6). *P < 0.0001 *vs.* siRd. The down-regulation of SMPX by siNOR-1 was also analyzed by Western blot (lower panel). Levels of β-actin were used as a loading control. (**b**) HSMM were differentiated to myotubes, and then were transfected with siNOR-1 or siRd. NOR-1 and SMPX mRNA levels (upper panel) and protein levels (lower panel) were analyzed as indicated in (**a**) (n = 6). *P < 0.0001, and ^#^P = 0.0002 *vs.* siRd.

**Figure 4:**
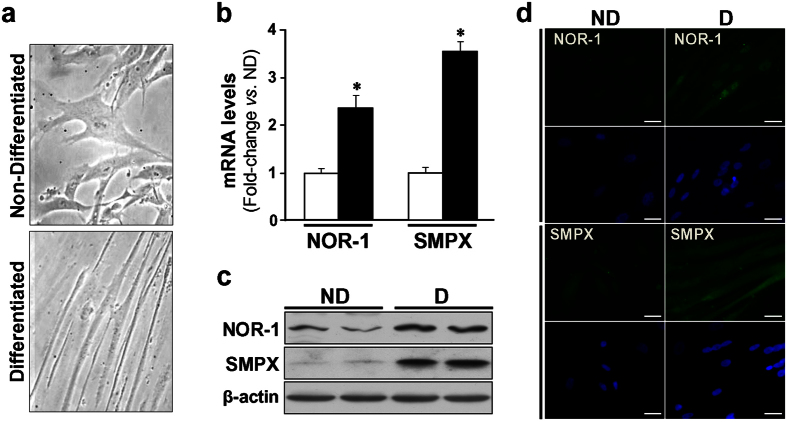
HSMM differentiation up-regulates the expression of NOR-1 and SMPX. (**a**) Microphotographs from HSMM maintained in normal growth medium (Non-differentiated; ND) or exposed to differentiation medium (Differentiated; D) to induce myotube formation. (**b**) mRNA levels of NOR-1 and SMPX in non-differentiated HSMM (white bars) and differentiated cells (black bars) (n = 6). *P < 0.0001 *vs*. ND. (**c**,**d**) Protein levels of NOR-1 and SMPX analyzed by Western blot (**c**) and immunocytochemistry (**d**) in cells as indicated in (**a**). Levels of β-actin were used as a loading control in Western blot experiments. In (**d**), NOR-1 (green), SMPX (green) and nuclear staining (blue) are shown. Bars: 25 μm.

To evaluate the impact of NOR-1 knockdown on SMPX expression, a siRNA was used to silence NOR-1; however, the low expression levels of SMPX in primary VSMC cultures preclude this approach. Since SMPX was early identified as a gene preferentially expressed in striated muscle, we analyzed the expression level of SMPX in HSMM cultures. The expression of SMPX was about 800-fold higher in these cells than in primary human VSMC cultures, and further increased in HSMM differentiated to myotubes (Supplementary Fig. S5). NOR-1 silencing, that was more efficient in non-differentiated (Fig. 3a) than in differentiated cells (Fig. 3b), was associated with a concomitant reduction of SMPX expression, as determined by real-time PCR and Western blot. The differentiation (in 2% horse serum during five days) transformed HSMM with typical myoblast morphology to elongated and multinuclear myotubes (Fig. 4a). HSMM differentiation increased NOR-1 and SMPX mRNA (≈ 2.4-fold and ≈3.5-fold, respectively) and protein levels, as determined by Western blot and immunocytochemistry (Fig. 4b–d).

### The effects of NOR-1 on SMPX and HSMM differentiation are independent events

**Figure 5:**
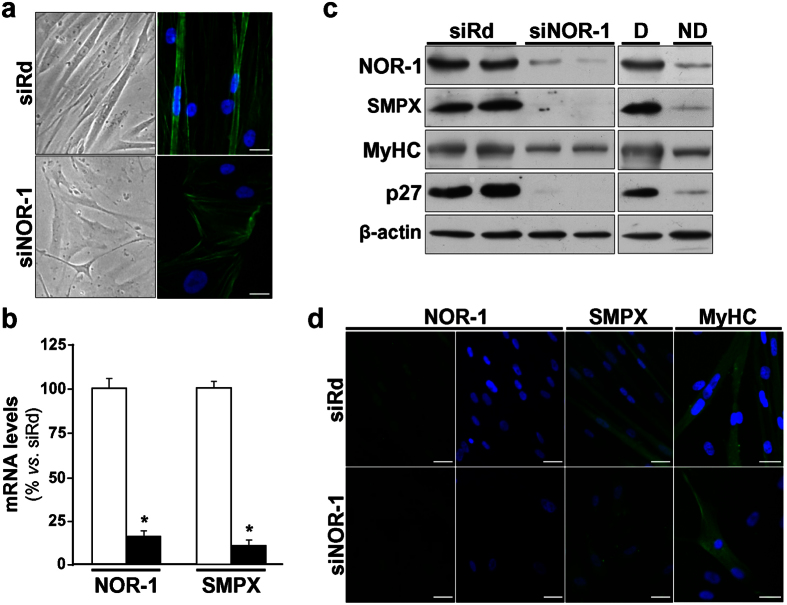
NOR-1 knockdown prevents HSMM differentiation and down-regulates SMPX expression. HSMM were transfected with a siRNA against NOR-1 (siNOR-1) or a control random siRNA (siRd), and then were exposed to differentiation conditions during five days. (**a**) Microphographs (left) and immunofluorescence microscopy analysis showing filamentous actin (F-actin stained with phalloidin; right) from HSMM treated as indicated above. (**b**) NOR-1 and SMPX mRNA levels in cells transfected with siNOR-1 (black bars) or with siRd (white bars) (n = 6). *P < 0.0001 *vs*. siRd. (**c**) Representative Western blot showing protein levels of NOR-1, SMPX, and cell markers associated to HSMM differentiation (MyHC and p27) in HSMM treated as indicated above. Levels of proteins in non-transfected HSMM, both differentiated (D) and non-differentiated (ND), are also shown. Levels of β-actin were used as a loading control (n = 4). (**d**) Immunofluorescence microscopy analysis showing NOR-1 (green), SMPX (green) and MyHC (green) in cells as indicated in (**a**). Nuclear staining using Hoechst (blue) is also shown. Bars: 25 μm.

**Figure 6:**
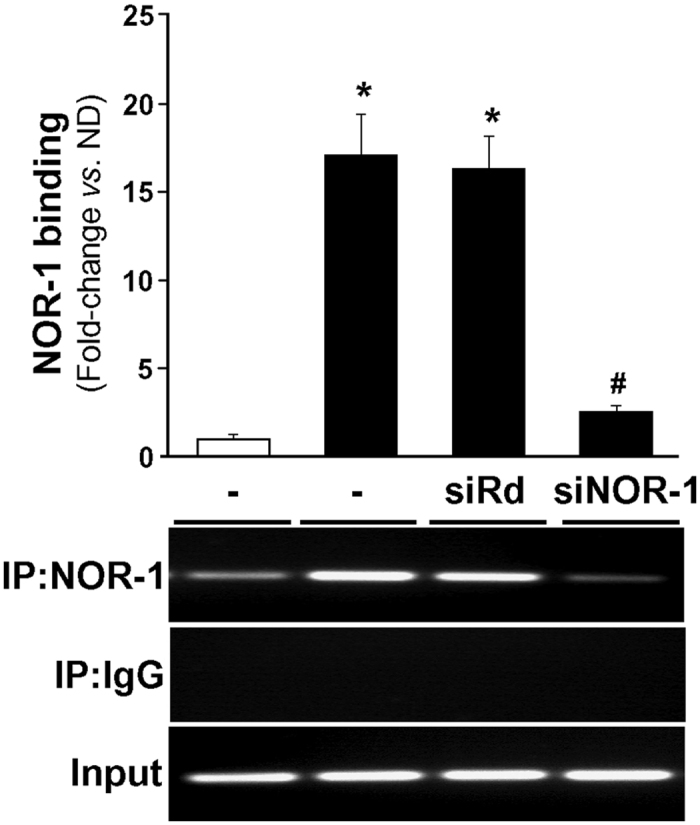
NOR-1 knockdown prevents the binding of NOR-1 to SMPX promoter. HSMM cells were transfected or not with a siRNA against NOR-1 (siNOR-1) or a control random siRNA (siRd), and then were exposed to differentiation conditions during five days (black bars). Non-differentiated HSMM were used as a control (white bars). The relative *in vivo* binding of NOR-1 to the human SMPX promoter was analyzed by ChIP assays. Immunoprecipitations were performed with an antibody against NOR-1 (IP:NOR-1) or a non-specific IgG (IP:IgG). The bars graph shows the levels enrichment of NOR-1 quantified by real-time PCR using SMPX promoter specific primers. Data were normalized to the total input DNA (n = 6). *P < 0.0001 *vs*. non-differentiated HSMM; ^#^P < 0.0001 *vs*. differentiated HSMM transfected or not with siRd. The bottom panel shows the agarose gel electrophoresis of PCR products. Equal input DNA and control IgG immunoprecipitations are shown.

**Figure 7:**
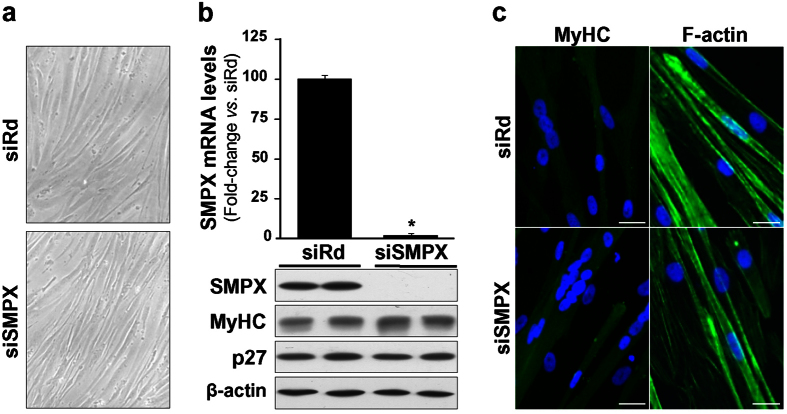
SMPX silencing does not affect HSMM differentiation. (**a**) Microphotographs from HSMM transfected with a siRNA against SMPX (siSMPX) or a control random siRNA (siRd) and then exposed to differentiation medium during five days. (**b**) SMPX mRNA levels (upper panel) and protein levels of SMPX, MyHC and p27 (lower panel) in HSMM treated as indicated in (**a**) (n = 6). *P < 0.0001 *vs*. siRd. Levels of β-actin were used as a loading control. (**c**) Immunocytochemistry for MyHC and phalloidin-stained F-actin in cells treated as indicated in (**a**). Bars: 25 μm.

NOR-1 silencing prevented the phenotypic changes as well as the up-regulation of SMPX (both mRNA and protein levels) associated to HSMM differentiation (Fig. 5). Interestingly, NOR-1 knockdown also prevented the up-regulation of cell differentiation markers such as MyHC and p27 (Fig. 5c,d). In ChIP assays, HSMM differentiation significantly increased the capacity of NOR-1 to bind to the NBRE(−167/−160) site in SMPX promoter, while this increase was abolished in cells previously transfected with a siRNA against NOR-1 (Fig. 6). SMPX silencing did not affect HSMM differentiation (Fig. 7). Thus, the NOR-1 mediated increase of SMPX expression associated to HSMM differentiation seems to be dispensable for cell differentiation.

## Discussion

NOR-1 exhibits a dissimilar expression pattern in different tissues^4^,^20^,^21^,^22^ and has been shown to regulate diverse and sometimes antithetical processes such as cell survival and apoptosis, and cell proliferation and differentiation^2^,^3^,^4^,^5^. Interestingly, the transient up-regulation of this immediate-early gene by cytokines and growth factors has been associated with VSMC proliferation and migration^4^,^14^,^15^,^16^,^17^,^18^, but in skeletal muscle, NOR-1 is induced by muscle contractile activity^28^,^29^,^30^ and has been related with muscle hypertrophy^23^,^24^. Since the role of NOR-1 in lipid and glucose metabolism was established, it has been proposed as a nexus that coordinates muscle performance and metabolic capacity^26^,^27^. Few NOR-1 target genes, however, have been described so far and none specifically related to the muscular tissue. Here we show that SMPX, a gene commonly expressed in striated muscle and identified as a stretch-responsive gene upregulated in skeletal muscle hypertrophy^31^, is a target of NOR-1. Further we assess the role of NOR-1 and SMPX in the differentiation of human myoblasts.

We used lentiviral vectors for stable ectopic expression of NOR-1 in human VSMC. The sustained over-expression of this early gene induced phenotypic changes in VSMC. Morphological and cell phenotypic changes are commonly accompanied by profound alterations in gene expression pattern affecting cytoskeleton and cytoskeletal-associated proteins. Since NOR-1 is a transcription factor and therefore modulates gene expression, as a first approach we analyzed mRNA levels of a set of genes previously associated with changes in cell morphology. Despite the observed reorganization of the actin filaments, neither the expression of β-actin nor that of smooth muscle specific α-SMA encoding genes experienced noticeable changes. Similarly, expression levels of several cytoskeletal associated proteins remained unchanged or experienced minor changes. By far the most striking result was the strong increase of SMPX, a gene typically expressed in both heart and skeletal muscle^32^.

SMPX (also called Chisel, Csl) is a small proline-rich protein (9 kDa) highly conserved among mammalian species^32^, that was firstly identified by reciprocal probing as a muscular protein selectively expressed in human cardiac and skeletal muscle cells^33^. By suppressive subtractive hybridization strategy, SMPX was later identified as a transcriptional target of the cardiac homeodomain factor Nkx2-5 in a mouse line carrying a null allele of Nkx2-5^32^, and as a stretch-responsive gene in mouse skeletal muscle^31^. Low SMPX expression, however, has also been detected in smooth muscle-rich tissues such as stomach^34^, intestine^32^,^34^ and pulmonary veins^32^. In embryos, SMPX is expressed in sub-regions of the developing heart corresponding to the future chamber myocardium and in developing skeletal muscles^35^. In fact, in the heart, SMPX is considered a chamber-specific gene, activated as part of a chamber myocardium-specific program of gene expression, which also includes Natriuretic precursor peptide type A (Nppa), Connexin (Cx) 40 and Cx43^32^,^36^. Interestingly, the formation of specialized heart structures such as the sinoatrial node (SAN) requires the local repression of these “chamber-specific” genes (including SMPX)^36^,^37^,^38^. Surprisingly, SMPX knockout mice showed no obvious phenotype; however, SMPX over-expression in the C2C12 myogenic cell line affect cytoskeletal dynamics and cell shape, and promote cell signaling-dependent myocyte fusion^32^,^35^. These results suggest that SMPX could play a role in the regulatory network through which muscle cells coordinate their structural and functional states during growth, adaptation, and repair^32^. However, the precise role played by SMPX in the musculature is presently unknown. Mutations affecting SMPX have been associated to x-chromosome linked hearing loss in humans^39^,^40^, but there is no information regarding SMPX in cardiovascular or muscular human disorders. Therefore, further studies are needed to understand the pathophysiological role of SMPX in human diseases.

As we mentioned above, SMPX is expressed in muscle tissues, predominantly in striated muscle and, in a lesser extent, in several smooth muscle tissues^31^,^33^,^34^. We confirmed this expression pattern using a panel of mouse tissues (Supplementary Fig. S6). In accordance with this expression pattern, previous studies of SMPX promoter identified putative *cis* regulatory elements for muscle-specific factors such as myocyte-specific enhancer factor (MEF2)^31^, a transcription factor involved in myogenesis^41^, MyoD^31^, a factor that triggers cell differentiation into myotubes, and Nkx-2.5^31^, a cardiac homeodomain transcription factor involved in cardiac development^42^. We also show for the first time that SMPX is expressed in the human vasculature (aorta and coronary arteries). Our *in silico* analysis revealed the presence of several putative NBRE sites potentially involved in the up-regulation of SMPX by NOR-1. We experimentally analyzed the functionality of these elements. Serial promoter deletion and site-directed mutagenesis analysis identified the proximal NBRE(−167/−160) site as crucial for the NOR-1-mediated induction of SMPX promoter activity. Accordingly, the direct binding of NOR-1 to this site was demonstrated *in vitro* and *in vivo* by EMSA and ChIP assays, respectively. Our results involving NOR-1 in the regulation of SMPX expand the knowledge on the SMPX promoter structure and regulation. The regulation of SMPX by NOR-1 was further confirmed in human skeletal muscle cells. SMPX mRNA levels were high in HSMM, and in the course of the differentiation to myotubes both NOR-1 and SMPX were up-regulated. NOR-1 silencing significantly reduced SMPX expression in both differentiated and non-differentiated HSMM. Interestingly, the differentiation process was dependent on NOR-1 but independent of SMPX. Although in certain contexts NR4A receptors (particularly NOR-1 and Nur77) can be totally or partially redundant^43^,^44^, which would hinder the observation of phenotypic consequences of deleting a single NR4A receptor, it should be emphasized that the specific silencing of NOR-1 prevented HSMM differentiation. Further studies are needed, however, to unravel the role of NOR-1 in the process of HSMM differentiation.

In summary, our data show that long lasting over-expression of NOR-1 in VSMC led to a non-proliferative, hypertrophic phenotype and to the induction of a protein commonly expressed in striated muscle whose expression further increases in skeletal muscle hypertrophy. Although the role of NOR-1 in hypertrophy remains to be elucidated, these results are coherent with the negative regulation of myostatin (anti-hypertrophic protein)^45^ by NOR-1 in the mouse skeletal muscle cell line C2C12^23^ and in the muscle of Nur77-transgenic mice^44^. It should be noted, however, that our *in silico* analysis identify the NBRE responsive for NOR-1 regulation in different mammalian species but not in murine rodents, and our experimental results discard SMPX as a NOR-1 target gene in mouse. Thus, unfortunately further testing on the functional significance of SMPX modulation by NOR-1 by using small animal models is not possible. Previous studies from us and others indicate that there are notable differences between human and murine rodents in both the mechanisms that regulate NOR-1^46^ and NOR-1 target genes^47^. Consequently, before extrapolate biomedical consequences from NOR-1 biology in mouse models, it is mandatory to corroborate the data in humans. Therefore, NOR-1 seems to complement other muscle regulatory factors in the regulation of proteins and processes in the musculature^26^, although further studies are required to establish the relevance of this nuclear receptor in the skeletal muscle biology in humans.

## Materials and Methods

### Human tissue sampling and preservation

The study was approved by the Hospital de la Santa Creu i Sant Pau (HSCSP) Ethics Committee and was conducted according to the Declaration of Helsinki. Written informed consent was obtained from each patient. Coronary arteries and aorta were collected from patients undergoing heart transplant and coronary artery at the HSCSP, and skeletal muscle samples were obtained from patients undergoing lower limb amputation at the same hospital. Specimens were snap-frozen in liquid nitrogen and stored at −80 °C until the preparation of protein and RNA extracts.

### Cell cultures

VSMC were obtained from human non-atherosclerotic arteries of hearts removed in transplant surgeries at the HSCSP by a modification of the explants technique^48^. All the procedures were approved by the Reviewer Institutional Committee on Human Research of the HSCSP and conform to the Declaration of Helsinki. Written informed consent was obtained from each patient. VSMC were cultured as described^14^. HSMM (Lonza) were cultured following the supplier’s recommendations. For HSMM differentiation, cells were cultured during five days in DMEM/F-12 supplemented with 2% horse serum, 2.5 mM L-glutamine, 15 mM HEPES, and 1 mM sodium pyruvate. The HEK293T and HeLa cell lines were cultured in DMEM supplemented with 10% FCS, 2 mM L-glutamine and antibiotics.

### Generation of lentiviral particles and transduction

The human NOR-1 cDNA was obtained from a pBlueScript-NOR1 construct, kindly provided by Dr. N. Ohkura (National Cancer Center Research Institute, Tokyo, Japan)^20^, and the mouse NOR-1 cDNA linked to a FLAG sequence was obtained from the plasmid pCMV5/NOR-1-FLAG, kindly provided by Dr. J. Hastie (University of Dundee, Scotland)^49^. The pLVX/NOR-1, pLVX/NOR-1-FLAG and pLVX/EGFP were obtained as described^10^. These constructs and the empty pLVX-Puro vector (pLVX) were transfected in HEK293T cells according to Lenti-X^TM^ Lentiviral Expression System Kit (Clontech). After 48 h, supernatants containing viral particles were harvested and titrated. Lentiviral transductions of VSMC were performed for 48 h at a multiplicity of infection of 15 in presence of polybrene (8 μg/mL), and transduced cell populations were enriched by puromycin selection (1.2 μg/mL). pLVX and pLVX/EGFP vectors were used as controls obtaining similar results.

### Analysis of cell proliferation and hypertrophy

The proliferation rate of lentiviral transduced and non-transduced VSMC was determined by cell counting. Cells were harvested by trypsinization, resuspended in growth medium and cell number was determined by using a Neubauer chamber. Data are expressed as the ratio of cell number/initial cell number. To determine total cell protein, trypsinized cells were washed with PBS, resuspended in lysis buffer (10 mM Tris/HCl pH 7.4, 1 mM Na_3_VO_4_, 0.5% SDS) and protein concentration was determined by the BCA Protein Assay™ (Pierce). The ratio of total protein/cell number was referred to that of non-transduced cells. Cell surface area was determined in subconfluent cell monolayers. Images were taken from five random fields and cell surface area was measured by using ImageJ 1.49 software (National Institute Health, USA). Data (arbitrary units) are expressed relative to those of non-transduced cells.

### Transfections with small interfering RNA (siRNA)

Silencer Select Pre-designed siRNA (Ambion) were used for NOR-1 (s15541) and SMPX (s22597) knockdown. The Silencer Select Negative Control #1 was used as a control. Briefly, one day before transfection, HSMM were seeded in complete growth medium. Then, the medium was replaced with antibiotic-free medium and cells were transfected for 8 hours with 20 nM siRNA, using 7.5 μL of Lipofectamine RNAiMAX Reagent (Invitrogen). After transfection, the medium was replaced with fresh antibiotic-free medium and cells were incubated for 48 h. Transfections of differentiated HSMM were performed in the same conditions, but cells were previously cultured in differentiation medium during five days and, after transfection, medium was replaced by fresh differentiation medium.

### Gene expression: real-time PCR

Total RNA was extracted from cell cultures using TRIsure^TM^ (Bioline) according to the manufacturers’ protocols. Total RNA (1 μg) was reverse-transcribed with the High Capacity cDNA Reverse Transcription Kit (Applied Biosystems). Levels of mRNA were assessed by real-time PCR using TaqMan^TM^ gene expression assays-on-demand (Applied Biosystems). TATA-binding protein (TBP) were used as endogenous controls^19^.

### SMPX promoter constructions and mutagenesis

A 2324 bp DNA fragment of the human SMPX promoter (nucleotides −2260 to +64 relative to the transcription start site) was generated by PCR and cloned into pGL3 vector (pGL3/SMPX-2260). The primers used were: 5′-TTATAGCTAGCCAGGAGTGCGGTATTGA-3′ (forward; *Nhe*I site is underlined) and 5′-TGAGCTGAGATCTCAATTCCGATGCT-3′ (reverse; *Bgl*II site is underlined). Promoter deletion constructs were generated by internal digestion with *Kpn*I (pGL3/SMPX-354) or by PCR using the reverse primer indicated above and the following forward primer: 5′-GCTTTGAGCTCGGTCAAGCCTTTCGGA-3′ (pGL3/SMPX-143; *Sac*I site is underlined). The NBRE(−167/−160) site present in SMPX promoter was mutated using the QuikChange^TM^ II Site-Directed Mutagenesis Kit (Stratagene) according to the manufacturer’s instructions. The primers used were: forward 5′-AGCCACCTCCCGGCTGctATTTGAGCCTGCTTTC-3′, and reverse 5′-GAAAGCAGGCTCAAATagCAGCCGGGAGGTGGCT-3′ (NBRE site is underlined and changes are indicated in lower case letters). *In silico* analysis of the mutated sequence confirm that no new response elements were introduced. Wild-type and mutated sequences were confirmed by DNA sequencing.

### Transient transfection and luciferase assays

Subconfluent VSMC were transfected using Lipofectamine LTX^TM^ and Plus Reagent (Invitrogen) according to the manufacturer’s instructions. SMPX constructs were co-transfected together with pCMV5/NOR-1 expression plasmid or the corresponding empty vector (pCMV5). Briefly, transient transfections were performed in 12 well-plates using 1.25 μL/well of Lipofectamine, 0.5 μL/well of Plus Reagent, 0.5 μg/well of SMPX construct, 0.05 μg/well of expression plasmid, and 0.05 μg/well of pRL-SV40 as an internal control. The DNA/liposome complexes were added to the cells for 16 h and then, cells were washed once and maintained in growth medium without antibiotics for 24 h. Luciferase activity was determined in cell lysates using the Dual-Luciferase^TM^ Reporter Assay System (Promega) according to the manufacturer’s protocol. Results were expressed as the ratio of firefly to renilla activity.

### Western blot analysis

Cellular extracts were obtained using a lysis buffer containing 10 mM Tris/HCl pH 7.4, 1 mM Na_3_VO_4_ and 0.5% SDS. Protein extracts from human tissues were generated using an ice-cold lysis buffer containing 50 mM Tris-HCl pH 7.5, 1% (v/v) Triton X-100, 150 mM NaCl and 1 mM DTT, supplemented with phosphatase and protease inhibitors. The protein concentration was determined by the BCA Protein Assay^TM^. Lysates were resolved by SDS-PAGE under reducing conditions and electrotransfered onto Immobilon polyvinylidene difluoride membranes. The membranes were probed using antibodies against FLAG epitope (F1804, Sigma-Aldrich), NOR-1 (sc-22519, Santa Cruz Biotechnology), SMPX (AV41597-100UG, Sigma-Aldrich), l-Cad (ab32330, Abcam), MYH10 (ab684, Abcam), MyHC (sc-20641, Santa Cruz Biotechnology), p27 (610242, BD Transduction Laboratories), and β-actin (A5441, Sigma-Aldrich), followed by appropriate horseradish peroxidase-conjugated secondary antibodies and a chemiluminiscent detection system. Equal loading of protein in each lane was verified by Ponceau staining and by β-actin levels.

### EMSA

Nuclear extracts were obtained from human VSMC by a modified Dignam method^19^. EMSA was performed as described using 5 μg of nuclear extracts^50^. Double-stranded DNA probes containing the putative wild-type NBRE(−167/−160) site in SMPX promoter (5′-CTCCCGGCTGACATTTGAGCCTGCTTTC-3′) and its mutated form (5′-CTCCCGGCTGctATTTGAGCCTGCTTTC-3′) were used (changes are indicated in lower case letters). DNA probes were labelled with [γ-^32^P]-ATP using T4 polynucleotide kinase and purified on a Sephadex G-50 column. In competition assays, the unlabeled probe was added before the labeled one and was incubated for 10 min on ice. For supershift assays, nuclear extracts were preincubated for 25 min with 2 μg of an anti-FLAG antibody (F1804) before adding the radiolabeled probe. Protein-DNA complexes were resolved by electrophoresis at 4 °C on 5% polyacrylamide gels in 0.5X TBE. Gels were dried and subjected to autoradiography using a Storage Phosphor Screen (GE Healthcare). Shifted bands were detected using a Typhoon 9400 Scanner (GE Healthcare).

### ChIP assay

VSMC or HSMM were cross-linked with 1% formaldehyde for 10 min. The cross-link reaction was stopped by adding glycine (100 mM). Then, cells were extensively washed and lysed in lysis buffer (LB) 1 (50 mM HEPES-KOH, pH 7.5, 140 mM NaCl, 1 mM EDTA, 0.5% NP-40, 0.3% Triton X-100, 10% glycerol), supplemented with protease inhibitors. After washing, nuclei were collected and resuspended in LB2 (10 mM Tris-HCl, pH 8.0, 100 mM NaCl, 1 mM EDTA, 0.5 mM EGTA, 0.1% Na-Deoxycholate, 0.5% N-lauroylsarcosine). Chromatin was sheared by sonication and an aliquot was saved and stored as input DNA. Supernatants were then immunoprecipitated with 5 μg of anti-FLAG antibody (F1804; for transduced VSMC), NOR-1 (sc-22519; for HSMM) or an IgG as a control. Immune complexes were recovered by addition of A/G-Agarose beads. After washing, complexes were extracted, cross-link was reversed and the DNA was purified and concentrated. Purified DNA was analyzed by conventional and real-time PCR with primers designed to amplify a 167 bp DNA fragment (forward primer: 5′-CGAACTTTGCTATACCCACTCA-3′; reverse primer: 5′-GTTTGATACAAGCCATACGCTG-3′). Real-time PCR from two independent experiments was performed by triplicate with the Quantifast^TM^ SYBR^TM^ Green PCR Kit (QIAGEN). Quantifications were analyzed by the ΔΔCt method and corrected to account for 4% inputs^51^.

### Immunocytochemistry

Cells were fixed in ice-cold 4% paraformaldehyde. After blocking, cells were incubated with anti-FLAG (F1804), anti-NOR-1 (H00008013-M06, Abnova), anti-SMPX (PA3-070, Pierce) or anti-MyHC (sc-20641) antibodies in PBS containing 5% BSA, overnight at 4 °C. Alexa Fluor 488 goat anti-rabbit and Alexa Fluor 488 goat anti-mouse immunoglobulins (Molecular Probes) were used as secondary antibodies. For nuclei and F-actin detections, Hoechst 33342 trihydrochloride trihydrate (H3570, Invitrogen) and Alexa Fluor phalloidin (A22284 (red), A12379 (green), Invitrogen), respectively were used. Controls without the primary antibody were included in all procedures. After extensive washing, cells were mounted with ProLong^TM^ Mounting Medium (Molecular Probes) and analyzed by confocal microscopy.

### Statistical analysis

Data are expressed as mean ± s.d. (unless otherwise state). Significant differences were established by Student’s t-test or one-way ANOVA, according to the number of groups compared, using the GraphPad Instat program (GraphPad Software V2.03) (GraphPad Software Inc.). Differences were considered significant at P < 0.05.

## Additional Information

**How to cite this article**: Ferrán, B. *et al*. The nuclear receptor NOR-1 regulates the small muscle protein, X-linked (SMPX) and myotube differentiation. *Sci. Rep.* **6**, 25944; doi: 10.1038/srep25944 (2016).

## Supplementary Material

Supplementary Information
