# Supplementary material for: The nuclear receptor NOR-1 regulates the small muscle protein, X-linked (SMPX) and myotube differentiation
Source: Sci Rep. 2016 May 16;6:25944. doi: 10.1038/srep25944 (PMC4867575; doi:10.1038/srep25944)
Supplement: Supplementary Information [file srep25944-s1.pdf]

Supplementary Information

The nuclear receptor NOR-1 regulates the small muscle protein, X-linked (SMPX) and myotube differentiation

Beatriz Ferrán[1], Ingrid Martí-Pàmies[1], Judith Alonso[1], Ricardo Rodríguez-Calvo[1], Silvia Aguiló[1], Francisco Vidal[2], Cristina Rodríguez[1]* & José Martínez-González[1]*

[1]Centro de Investigación Cardiovascular, CSIC-ICCC, IIB-Sant Pau, c/Sant Antoni Maria Claret 167, 08025 Barcelona, Spain. [2]Unitat de Diagnòstic i Teràpia Molecular, Banc de Sang i Teixits, Passeig Vall d'Hebron 119-129, Barcelona 08035, Spain.

* The last two authors contributed equally to this work

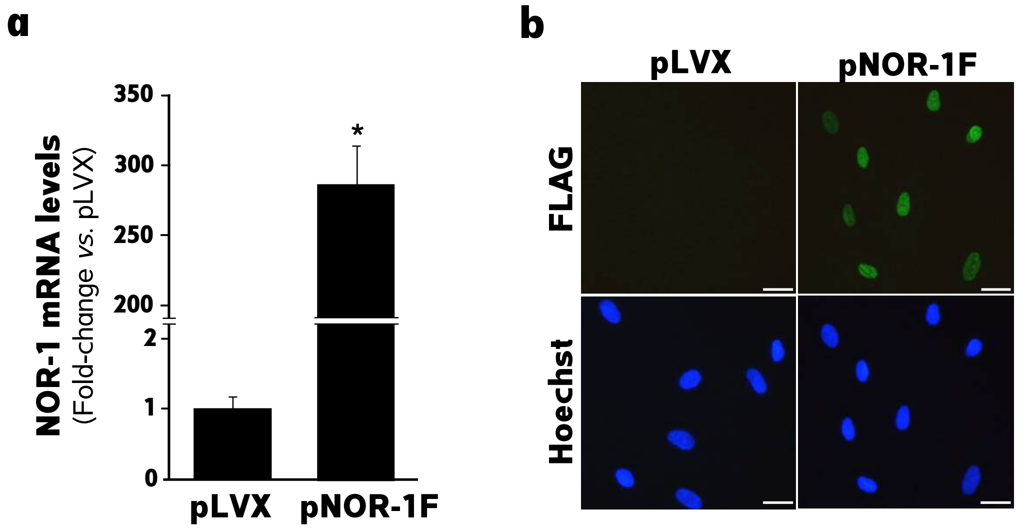

Supplementary Fig. S1. Lentiviral over-expression of NOR-1 in human VSMC. (**a**) Human VSMC were transduced with pLVX or pLVX/NOR-1-FLAG (pNOR-1F) lentivirus, and NOR-1 over-expression was verified by real-time PCR (n = 6). *P < 0.0001 *vs.* cells transduced with pLVX. (**b**) Immunocytochemical analysis for NOR-1 using an antibody against the FLAG sequence (FLAG), from cells transduced as indicated in (a). Bottom panels: fluorescent labelling of nuclei using Hoechst. Bars: 25 μm.

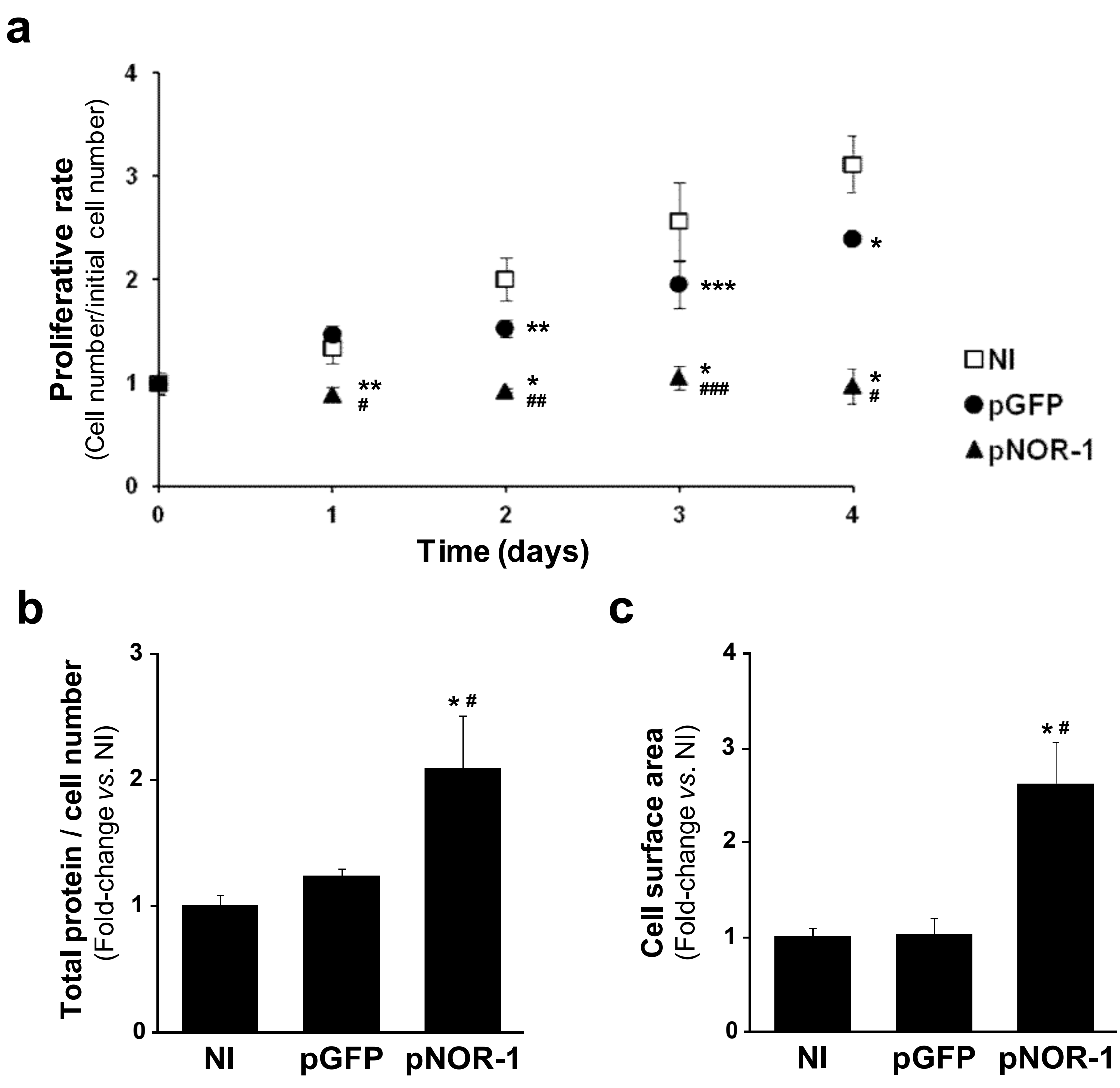

Supplemetary Fig. S2. Lentiviral NOR-1 over-expression reduces VSMC proliferative rate and promotes VSMC hypertrophy. (**a**) VSMC were transduced with a lentiviral vector to over-express NOR-1 (pLVX/NOR-1; pNOR-1), or a control lentiviral vector expressing EGFP (pLVX/EGFP; pGFP), and the proliferative rate of VSMC cultures was determined by daily cell counting. Non-infected cells (NI) were used as a control. (**b** and **c**) Relative cellular protein content (total protein content/ cell number) (**b**) and relative cell surface area (**c**) of VSMC treated as indicated in (a). *, **, ***, P < 0.0001, P = 0.002, P = 0.0077, respectively *vs*. NI. #, ##, ###, P < 0.0001, P = 0.0003, P = 0.0006, respectively *vs*. pGFP.

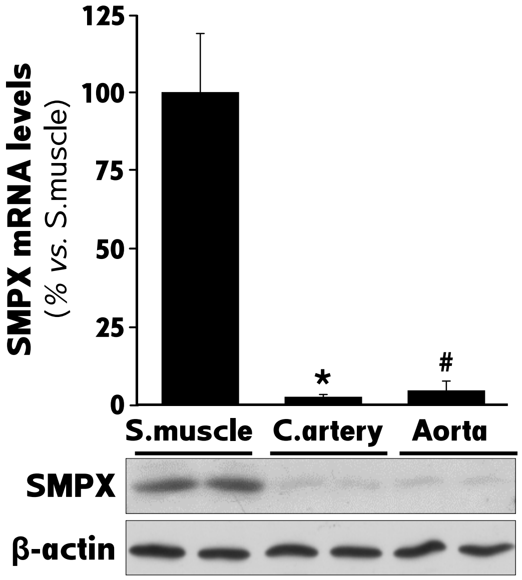

Supplementary Fig. S3. **SMPX is expressed in human arteries**. (**a**) SMPX mRNA levels determined by real-time PCR in human skeletal muscle (S. muscle; n = 4), and human vessels: coronary arteries (C. artery; n = 7) and aorta (n = 6). Data are expressed as mean ± s.e.m. *P = 0.0001 and #P = 0.0003 *vs.* S. muscle. (**b**) Representative Western blot showing SMPX protein levels in these tissues. Levels of β-actin were used as a loading control in Western blot experiments.

a

```
                          NBRE c.s.  TGACCTTT
                                     |||| |||
Human       AAGTAAGATACCATAGCCACCTCCCGGCTGACATTTGAGC-CTGCTTTCCGCACGGTCAAGCCT
Orangutan   AAGTAAGATACCATAGCCACCTCCCGGCTGACATTTGAGC-gcttTccgCaCggtcaggccttT
Chimpanzee  AAGTAAGATACCATAGCCACCTCCCGGCTGACATTTGAGC-gcttTccgCaCggtcaagccttT
Gorilla     AAGTAAGATACCATAGCCACCTCCCGGCTGACATTTGAGC-gcttTccgCaCggtcaagccttT
Cow         A-GTAAGATtCCAgAGCCACCTtCCGGCTGACATTTGAGCcCTGCTTTCaGCACGGTCAAGCCT
Pig         A-GTAAGATtCCgTAGCCACCTtCCGGCTGACATTTGAGCcCTGCTTTCaGCACGGTCAAGCCT
Mouse       A-GaAAGATAaCgTAGCCACCTCCaGGCTGAtATcTGAGCcCTGCTTTCCaCACGGTCAAGCCT
Rat         A-GTAAGATAaCgTAGCCACCTCCacGCTGAtATcTGAGCcCTGCTTTCCaCAtGGTCAAGCCT
```

b **c**

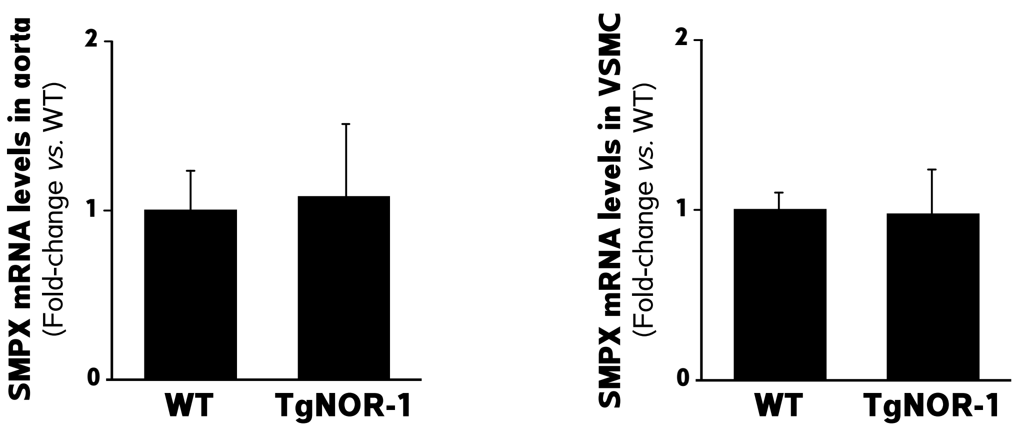

Supplementary Fig. S4. SMPX does not seem to be regulated by NOR-1 in mouse. (**a**) Alignment of the proximal region corresponding to the SMPX promoter from different species including human and mouse. Non-conserved positions are indicated in lower case. The NBRE consensus sequence (NBRE c.s.) is shown at the top. (**b** and **c**) SMPX was not regulated by NOR-1 transgenesis in mice. SMPX mRNA levels were analyzed by real-time PCR in mouse aorta from wild-type (WT; n = 7) and transgenic mice that specifically over-express human NOR-1 in VSMC (TgNOR-1; n = 9) (b), and in cultures of VSMC from these animals (n = 6) (c). Data are expressed as mean ± s.e.m.

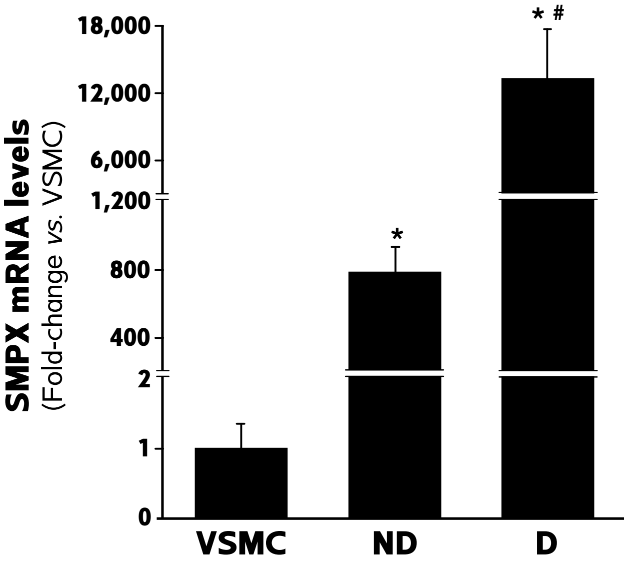

Supplementary Fig. S5. SMPX is highly expressed in HSMM. mRNA levels of SMPX (analysed by real-time PCR) in human VSMC, and in HSMM non-differentiated (ND) and differentiated (D) to myotubes (after the exposition to 2% horse serum during five days) (n = 6). *$P < 0.0001$ *vs.* VSMC; #$P < 0.0001$ *vs.* ND.

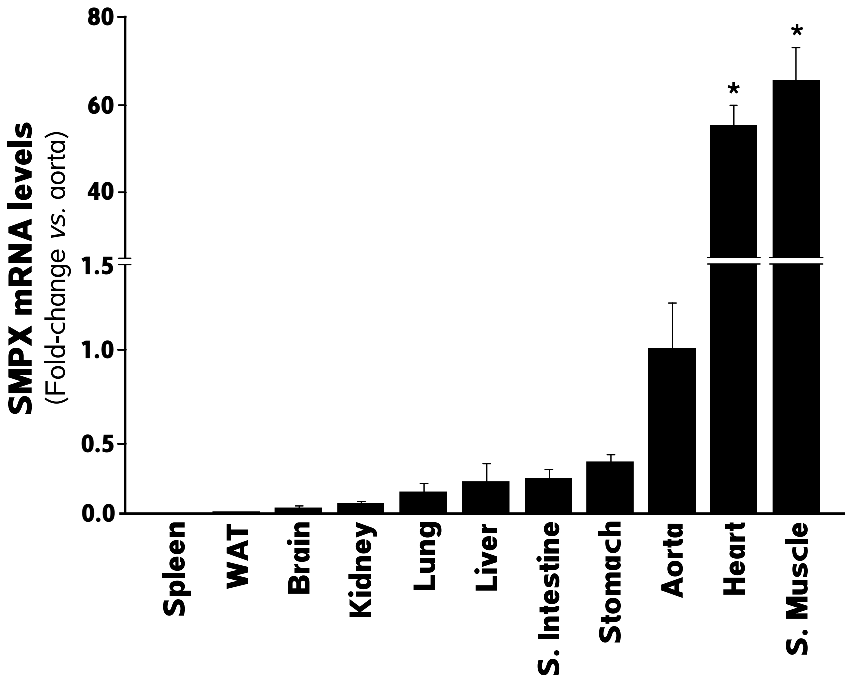

Supplementary Fig. S6. SMPX expression in mouse tissues. Expression levels of SMPX analysed by real-time PCR in a set of mouse tissues. WAT: white adipose tissue; S. intestine: small intestine; S. muscle: skeletal muscle. Data are expressed as mean ± s.e.m. (n = 7). *P < 0.0001 *vs.* aorta.
